# Poor sleep quality may trigger cognitive deficits after recovery from COVID-19

**DOI:** 10.3389/fpsyg.2024.1382875

**Published:** 2024-05-27

**Authors:** A. Carnes-Vendrell, G. Piñol-Ripoll, M. Ariza, N. Cano, B. Segura, C. Junque, J. Béjar, C. Barrue, M. Garolera, Nautilus Project Collaborative Group, Vanesa Arauzo, Jose A. Bernia, Marta Balague-Marmaña, Berta Valles-Pauls, Jesús Caballero, Ester Gonzalez-Aguado, Carme Tayó-Juli, Eva Forcadell-Ferreres, Silvia Reverte-Vilarroya, Susanna Forné, Anna Bartes-Plans, Jordina Muñoz-Padros, Jose A. Muñoz-Moreno, Anna Prats-Paris, Inmaculada Rico, Nuria Sabé, Marta Almeria, Laura Casas, Maria José Ciudad, Anna Ferré, Tamar Garzon, Manuela Lozano, Marta Cullell, Sonia Vega, Sílvia Alsina, Maria J. Maldonado-Belmonte, Susana Vazquez-Rivera, Eva Baillès, Sandra Navarro, Ayoze González Hernández, Yaiza Molina, Victoria Olive, Silvia Cañizares

**Affiliations:** ^1^Cognitive Disorders Unit, Cognition and Behavior Study Group, Hospital Universitari Santa Maria, Lleida, Spain; ^2^Clinical Research Group for Brain, Cognition and Behavior, Consorci Sanitari de Terrassa, Terrassa, Spain; ^3^Departament de Ciències Bàsiques, Universitat Internacional de Catalunya (UIC), Barcelona, Spain; ^4^Medical Psychology Unit, Department of Medicine, Universitat de Barcelona, Barcelona, Spain; ^5^Institute of Neurosciences, University of Barcelona, Barcelona, Spain; ^6^Institut d’Investigacions Biomèdiques August Pi i Sunyer (IDIBAPS), Barcelona, Spain; ^7^Centro de Investigación Biomédica en Red sobre Enfermedades Neurodegenerativas (CIBERNED), Barcelona, Spain; ^8^Faculty of Informatics of Barcelona (FIB), Polytechnic University of Catalonia, Barcelona, Spain; ^9^Neuropsychology Unit, Consorci Sanitari de Terrassa, Terrassa, Spain

**Keywords:** cognition, cognitive reserve, COVID-19, fatigue, post-COVID-19 condition, quality of life, sleep quality, subjective cognitive complaints

## Abstract

**Objective:**

In the present study, we aimed to assess the cognition of post-COVID-19 condition (PCC) participants in relation to their subjective sleep quality (Pittsburgh Sleep Quality Index, PSQI) and to analyse possible moderators of this effect, such as quality of life (European Quality of Life-5 Dimensions, EQ-5D), fatigue (Chadler Fatigue Questionnaire, CFQ), cognitive reserve (Cognitive Reserve Questionnaire, CRC), and subjective cognitive complaints (Memory Failures of Everyday Questionnaire, MFE-30).

**Methods:**

We included 373 individuals with PCC and 126 healthy controls (HCs) from the NAUTILUS Project (NCT05307549 and NCT05307575) who were assessed with a comprehensive neuropsychological battery and various questionnaires.

**Results:**

We found that PCC participants with poor sleep quality had a 4.3% greater risk of immediate verbal memory deficits than those with good sleep quality, as indicated by the greater odds ratio (OR) of 1.043 and confidence interval (CI) of 1.023–1.063. Additionally, their risk of immediate verbal memory disorders was multiplied by 2.4 when their EQ-5D score was low (OR 0.33; CI 0.145–0.748), and they had a lower risk of delayed visual memory deficits with a greater CRC (OR 0.963; CI 0.929–0.999). With respect to processing speed, PCC participants with poor sleep quality had a 6.7% greater risk of deficits as the MFE increased (OR 1.059; CI 1.024–1.096), and the risk of slowed processing speed tripled with a lower EQ-5D (OR 0.021; CI 0.003–0.141).

**Conclusion:**

These results indicate that poor subjective sleep quality is a potential trigger for cognitive deficits. Therapeutic strategies to maximize sleep quality could include reducing sleep disturbances and perhaps cognitive impairment in PCC individuals.

## 1 Introduction

Cognition in post-COVID-19 condition (PCC) participants has been widely described. According to Soriano et al., PCC is characterized by a wide variety of symptoms that can be fixed or fluctuating, manifest 3 months after the onset of the disease, persist for at least 2 months, and cannot be explained by other diseases (Soriano et al., 2022). Studies have shown that 60 to 80% of PCC patients experience brain fog and impairment in several cognitive domains, such as attention, processing speed, memory, and executive function (Davis et al., 2021; Ariza et al., 2022; Delgado-Alonso et al., 2022; García-Sánchez et al., 2022; Guo et al., 2022; Matias-Guiu et al., 2022; Ziauddeen et al., 2022). A review revealed that deficits in global cognitive function in people with PCC range from 15 to 80% (Daroische et al., 2021), which demonstrates the heterogeneous results in the field. Several studies have compared the severity of disease among PCC patients using comprehensive neuropsychological tests. Some have shown differences between nonhospitalized and hospitalized patients, with the latter exhibiting greater impairment in attention, executive function, and processing speed (Becker et al., 2021; García-Sánchez et al., 2022; Santoyo-Mora et al., 2022; Vannorsdall et al., 2022; Ariza et al., 2023a). In contrast, one systematic review concluded that outpatients were more likely than hospitalized patients to have cognitive deficits (Premraj et al., 2022).

Poor sleep quality has also been described in hospitalized and nonhospitalized COVID-19 patients (Akıncı and Melek Başar, 2021; Al-Ameri et al., 2022; Karimi et al., 2022; Malik et al., 2022; Samushiya et al., 2022). The prevalence of sleep disturbances ranges from 57 to 74.8% (Alimoradi et al., 2021; Jahrami et al., 2021), which makes sleep disturbances one of the most prevalent symptoms in PCC patients. Few studies have differentiated the effects of sleep quality on individuals with PCC with respect to disease severity. Chhajer and Shukla (2022) showed that patients in the severe group [those admitted to the intensive care unit (ICU)] had poorer quality of sleep. However, in our recent study, sleep quality was significantly worse in the COVID-19 group than in the healthy control group when we studied a large sample of participants, but no differences were found with regard to severity (mild, hospitalized or ICU patients). In the same study, we found that the prevalence of poorer sleep quality was also significantly greater in the COVID-19 group (Carnes-Vendrell et al., 2024).

Sleep and cognition are closely related. The sleep–wake cycle is regulated by complex interactions among brain regions and neurotransmitter systems, and many of these interactions are implicated in cognitive functions (Diekelmann and Born, 2010; Lim and Dinges, 2010). Certain aspects of sleep, such as slow-wave sleep, appear to have effects on the performance of the prefrontal cortex (PFC), which in turn may affect cognitive processes that depend on the PFC (Muzur et al., 2002; Wilckens et al., 2012, 2014). Thus, executive functions, which are supported by the PFC, could be more sensitive to sleep.

The role of sleep quality in cognition has been widely studied in older adults because of its implications for neurodegenerative diseases. However, the relationship between sleep complaints and worse cognitive performance in this population is not consistent. Some authors have shown that reduced sleep quality is associated with an increased risk of cognitive decline or dementia (Jelicic et al., 2002; Potvin et al., 2012; Sterniczuk et al., 2013). In this regard, previous studies have reported that poorer quality of sleep is related to worse performance in several executive functions (Nebes et al., 2009), verbal memory and visuospatial reasoning (Schmutte et al., 2007). Nevertheless, previous findings are not conclusive since not all studies have related sleep quality to the same cognitive domain.

Despite the extensive literature on the influence of COVID-19 sequalae on cognition and sleep quality, few studies have analysed this relationship. The majority of studies did not find a relationship between these two variables and only reported a relationship between cognition and other variables, such as fatigue, quality of life and depression (Bungenberg et al., 2022; Margalit et al., 2022; Bolattürk and Soylu, 2023; Ozdemir and Tastemur, 2023). Two studies did show an association with cognition and sleep, but only with daytime sleepiness (Bungenberg et al., 2022; Schild et al., 2023). Hartung et al. (2022) found that cognition and sleep quality were significantly related in a univariate analysis but not in a multivariate analysis. Although some associations between cognition and these variables have been found in previous studies, to our knowledge, none of them have attempted to analyse the relationship between sleep quality and cognition, including the possible moderating effects of these variables (e.g., fatigue, quality of life).

Therefore, the aim of this study was (i) to assess the cognition of PCC participants with regard to their sleep quality to determine whether good or bad sleep quality implies differences in cognition and (ii) to analyse different possible moderators of this effect, such as quality of life, fatigue, cognitive reserve, and daily memory failure.

## 2 Materials and methods

### 2.1 Participants

We included 499 participants from the Nautilus Project (ClincalTrials.gov IDs: NCT05307549 and NCT05307575), of whom 373 had post-COVID-19 conditions (PCC) and 126 were healthy controls (HCs). Of the PCC patients, 206 were nonhospitalized (mild PCC), 84 were hospitalized, and 83 were admitted to the ICU. Mild PCC only showed mild COVID-19 symptoms in the acute phase, while hospitalized and ICU PCC had severe complications that needed hospitalization, such as pneumonia. As this was a cross-sectional study, the sample was recruited across 16 hospitals in Spain and Andorra consecutively. It was coordinated by the Consorci Sanitari de Terrassa (Terrassa, Barcelona, Spain). Recruitment was carried out between June 2021 and October 2022.

The inclusion criteria for the PCC group were a confirmed diagnosis of COVID-19 according to the WHO criteria with signs and symptoms of the disease during the acute phase, a period of at least 12 weeks after infection, and age between 18 and 65 years. The exclusion criteria were an established diagnosis of a psychiatric disorder, neurological disorder, neurodevelopmental disorder, or systemic pathology known to cause cognitive deficits before COVID-19 infection and motor or sensory alterations that could interfere with the neuropsychological assessment. The HCs had not had COVID-19 infection (no positive tests or compatible symptoms). The same exclusion criteria for the PCC group were applied to the HC group.

### 2.2 Procedure

This procedure has been previously described in another study (Carnes-Vendrell et al., 2024). In summary, participation was completely voluntary, and we obtained written informed consent from all the participants before inclusion. We collected data on sociodemographic characteristics, previous comorbidities and COVID-19 symptoms in the first session. At the second visit, the neuropsychological assessment was performed. Different cognitive domains were assessed with an extensive and comprehensive neuropsychological battery. The Spanish version of Rey’s Auditory Verbal Learning Test (RAVLT) (Schmidt, 1996; Alviarez-Schulze et al., 2022) was used to assess verbal memory, whereas the Rey–Osterrieth Complex Figure Test (ROCF) (Meyers and Meyers, 1996) was used for visual memory (immediate and delayed). The copy trial of the ROCF evaluated visuoconstructive abilities. The WAIS-III Digit Span subtest was used to measure verbal attention (digit span forward) and working memory (digit span backward) (Wechsler, 1999). Information processing speed was assessed with the digit symbol (DS) test from the WAIS-III (Wechsler, 1999). Parts A and B of the Trail Making Test (TMT) were administered to measure visual scanning, motor speed and attention, and mental flexibility (Reitan, 1958). Verbal and semantic fluency were assessed with the Controlled Oral Word Association Test (COWAT) (Benton and Hamsher, 1989). However, for verbal fluency, the letters used were P, M and R because Spanish normative data exist for these three tests (Peña-Casanova et al., 2009). The third part of the Stroop (colour-word) test (Golden, 2005) was used as a measure of cognitive inhibitory control (executive functions). To evaluate language, the Boston Naming Test (BNT) was administered (Allegri et al., 1997). Finally, emotion recognition was assessed with the Reading the Mind in the Eye Test (Fernández-Abascal et al., 2013). In addition, information on the cognitive reserve of all participants was collected with the Cognitive Reserve Questionnaire (CRC) (González et al., 2011). All evaluations were performed by trained neuropsychologists.

The participants were given all of the questionnaires to complete online or on paper to assess different variables. In this study, we focused on sleep quality, which was assessed with the Pittsburgh Sleep Quality Index (PSQI) (Buysse et al., 1989); everyday memory failure, which was assessed with the Memory Failures of Everyday Questionnaire (MFE-30) (Sunderland et al., 1984); fatigue, which was assessed with the Chadler Fatigue Questionnaire (CFQ) (Jackson, 2015); and quality of life, which was assessed with the European Quality of Life-5 Dimensions (EQ-5D) (Foundation ER, 2018).

The participants’ anonymity and confidentiality were guaranteed. The Scientific Ethics Committee of the Hospital Universitari Arnau de Vilanova approved both the study and the consent procedure (CEIC 2384), as did the Drug Research Ethics Committee (CEIm) of Consorci Sanitari de Terrassa (CEIm code: 02-20-107-070) and the Ethics Committee of the University of Barcelona (IRB00003099). Additionally, the investigation was conducted in accordance with the latest version of the Declaration of Helsinki.

### 2.3 Statistical analysis

Descriptive analyses were performed to compare healthy controls with PCC patients. For categorical variables, frequencies and percentages were obtained, and for quantitative variables, the means and standard deviations were obtained. The cognition variables were converted into dichotomous variables depending on whether the result indicated cognitive impairment (−1 standard deviation from the mean), which is why it is shown as a percentage of impairment for each variable. PSQI score of 5 was used as a cutoff indicating good or bad subjective sleep quality (Buysse et al., 1989). Pearson’s nonparametric X^2^ test was conducted, and Fisher’s exact test was used for pairwise differences between groups only for 2×2 tables. Differences in continuous variables between groups were assessed using factorial ANOVA with a general linear model and powerful estimation (robust covariances). All multiple comparisons were adjusted by Bonferroni correction.

To study the relationship between sleep quality and cognitive impairment in PCC and to verify the possible moderating effect of other variables of interest (cognitive reserve, fatigue, subjective cognitive complaints and quality of life), binary logistic regression was applied for each cognitive variable (alteration vs. non-alteration). The explanatory variables were sleep quality (<= 5 good; >5 bad), the possible moderators (MFE-30, CRC, CFQ and EQ_5D) and the 4 interactions between moderators and sleep quality. The models were adjusted for sex, age, and years of schooling and were calculated for PCC participants and healthy controls separately. If the interaction was significant (*p* < 0.05), then moderation was calculated. Only results in which moderation was present are shown in the results section. The odds ratio (OR) is shown with the 95% CI. For the quality of fit, the Hosmer–Lemeshow test and the area under the curve (AUC) were used.

The statistical significance level used in the analyses was 5% (α = 0.05). All analyses were performed with IBM SPSS statistics 26.

## 3 Results

### 3.1 Characteristics of participants

Of the 373 PCC patients, 206 had mild PCC (the ones that had not been hospitalized) (mean age 46.74 years, standard deviation 9.47), 84 were hospitalized (53.07 ± 8.83), and 83 were admitted to the ICU (52.24 ± 8.36). In the mild PCC group, the majority of participants were female (70.1%), while in the hospitalized and ICU groups, the majority of participants were male (51.2 and 53%, respectively). Patients in the ICU-PCC group consumed more alcohol (39.8%), were more likely to be obese (54.2%) and had more previous comorbidities, such as high blood pressure (30.1%) and dyslipidaemia (22.9%), whereas the hospitalized PCC group had more chronic pain (17.3%) and diabetes mellitus (9.5%). Table 1 shows the clinical and sociodemographic characteristics of the sample.

**Table 1:** Clinical and sociodemographic characteristics of the sample.

	Healthy controls	Mild PCC	Hospitalized PCC	ICU PCC	*p* value
	*n* = 126	*n* = 206	*n* = 84	*n* = 83	<0.001***
Age (years) (SD)	46.33 (10.01)	46.74 (9.47)	53.07 (8.83)	52.24 (8.36)	<0.001***
Female (%)	73.8%	79.1%	48.8%	47.0%	<0.001***
Years of education (SD)	15.54 (2.99)	14.38 (3.21)	13.20 (3.47)	13.07 (3.18)	<0.001***
Days since COVID-19 (SD)	–	362.80 (201.44)	301.14 (146.86)	265.42 (113.03)	<0.001***
MoCA (SD)	27.90 (1.81)	26.11 (2.78)	25.58 (3.01)	25.08 (2.97)	<0.001***
BMI (SD)	25.03 (5.98)	25.48 (4.97)	27.80 (5.21)	31.27 (5.32)	<0.001***
Tobacco smoking (%)	24.6%	8.7%	4.8%	4.8%	<0.001***
Alcohol consumption (%)	28.6%	23.8%	28.6%	39.8%	0.060
**Civil status**					
Married (%)	69.0%	79.6%	72.6%	81.9%	0.033*
**Previous comorbidities**					
Heart disease (%)	2.4%	2.5%	3.6%	3.6%	0.903
Respiratory disease (%)	4.8%	13.1%	14.3%	15.7%	0.044*
Chronic kidney disease (%)	0.0%	1.0%	1.2%	1.2%	0.700
High blood pressure (%)	2.4%	7.8%	19.0%	30.1%	<0.001***
Dyslipidemia (%)	9.5%	9.2%	19.0%	22.9%	0.003**
Diabetes mellitus (%)	2.4%	0.5%	9.5%	7.2%	<0.001***
Obesity (%)	11.9%	17.0%	34.5%	54.2%	<0.001***
Chronic liver disease (%)	0.0%	0.5%	4.8%	3.6%	0.011*
Chronic pain (%)	4.0%	5.2%	17.3%	7.2%	0.002**
**Quality of sleep**					
PSQI total score (SD)	5.38 (3.22)	8.99 (4.06)	8.58 (4.69)	8.11 (454)	<0.001***
Poor quality of sleep (>5)	40.2%	77.2%	66.7%	65.1%	<0.001***

Unless otherwise specified, results are presented as mean (standard deviation). Level of statistical significance = *p < 0.05, **p < 0.01, ***p < 0.001. PCC, Post-COVID-19 Condition; MoCA, Montreal Cognitive Assessment; BMI, Body Mass Index. Bold values indicate statistical significance.

In terms of sleep quality, the PCC participants had worse sleep quality than the healthy control participants (*p* < 0.001). However, there were no statistically significant differences between the groups based on PCC severity. We found significant differences between the groups (*p* < 0.001) in the percentage of participants who reported poor quality of sleep (those who obtained ≥5 points on the PSQI). The healthy control group had a lower percentage of responses above 5 on the PSQI (40.2%) than the mild-PCC (77.2%) and hospitalized-PCC (66.7%) groups (Table 1).

Of the moderator variables, we found statistically significant differences between PCC patients and HCs in quality of life, daily memory failure, fatigue and cognitive reserve (Table 2). PCC patients obtained worse results for all of these variables.

**Table 2:** Description of different moderators in the sample.

	Healthy controls	Mild PCC	Hospitalized PCC	ICU PCC	*F*	*p* value
CRC	16.60 (3.31)	15.27 (3.75)	14.10 (4.51)	13.29 (4.68)	13.655,3,495	<0.001***
MFE-30	7.85 (6.71)	21.31 (11.36)	17.38 (13.65)	15.25 (11.62)	65.495,3,495	<0.001***
CFQ	1.86 (2.68)	7.80 (3.59)	5.15 (4.15)	5.61 (4.07)	100.713,3,495	<0.001***
EQ_5D	0.91 (0.12)	0.69 (0.23)	0.71 (0.22)	0.75 (0.18)	56.223,3,491	<0.001***

Results are presented as mean (standard deviation). Level of statistical significance = *p < 0.05, **p < 0.01, ***p < 0.001. PCC, Post-COVID-19 Condition; CRC, Cognitive Reserve Cuestionnaire; MFE-30, Memory Failures of Everyday Questionnaire; CFQ, Chadler Fatigue Questionnaire; EQ-5D, European Quality of Life-5 Dimensions. Bold values indicate statistical significance.

### 3.2 Cognitive performance according to PCC severity

Many differences in cognitive performance were found between PCC patients and healthy controls (Table 3). Participants with mild PCC had significantly greater impairment in executive function variables, such as Stroop colour-word (*p* = 0.006), verbal fluency (*p* = 0.012) and semantic fluency (*p* = 0.030), than HCs. However, compared with HCs, hospitalized PCC participants had worse verbal memory [learning (*p* < 0.001) and delayed recall (*p* = 0.002)], whereas ICU PCC patients had more deficits in verbal memory with immediate recall (*p* = 0.001) than the HC. In addition, these participants obtained higher percentages of alterations in attention and information processing speed tasks, such as the digit symbol (*p* = 0.018), TMT A (*p* = 0.015) and TMT B (*p* = 0.002), as well as in working memory (digit span forward, *p* = 0.018) and emotion recognition (*p* = 0.001).

**Table 3:** Cognitive performance according to PCC severity.

	Healthy controls	Mild PCC	Hospitalized PCC	ICU PCC	χ*^2^*	*p* value
	*n*=126	*n*=206	*n*=84	*n*=83		
Memory						
RAVLT_total score	17.5%	36.9%	42.9%	34.9%	19080.3	<0.001***
RAVLT_immediate recall	13.5%	29.1%	34.5%	34.9%	17,182.30	0.001**
RAVLT_delayed recall	12.7%	30.1%	31.0%	27.7%	14,707.30	0.002**
ROCF_immediate recall	18.0%	20.4%	21.0%	23.7%	0.942,3	0.815
ROCF_delayed recall	18.3%	23.3%	26.2%	31.3%	5,014.30	0.171
Attention and processing speed						
Digit span backward	21.4%	29.1%	26.2%	38.6%	7493.3	0.058
Digit symbol	0.8%	7.8%	7.2%	10.8%	10,060.30	0.018*
TMT_A	11.1%	19.9%	15.5%	20.5%	10,506.30	0.015*
TMT_B	11.1%	16.6%	17.3%	28.0%	14,476.30	0.002**
Executive functions						
Digit span forward	6.3%	17.0%	10.7%	19.3%	10,109.30	0.018*
Stroop_color word	12.1%	30.6%	25.0%	27.2%	12,481.30	0.006**
Verbal fluency_P	5.6%	16.5%	10.7%	20.5%	12,481.30	0.006**
Verbal fluency M	6.3%	10.7%	13.1%	13.3%	3,609.30	0.307
Verbal fluency R	5.6%	16.1%	11.9%	8.5%	9,327.30	0.025*
Verbal fluency_total PMR	0.8%	10.2%	6.0%	8.4%	10,968.30	0.012*
Language						
Semantic fluency	12.7%	25.2%	16.7%	24.1%	8,983.30	0.030*
BNT	6.3%	9.2%	8.3%	13.3%	2,967.30	0.397
Social cognition						
Eye Test	20.6%	28.2%	33.7%	45.7%	15,617.30	0.001**
Constructional praxis						
ROCF_copy trial	18.3%	22.3%	32.1%	28.9%	6,739.30	0.081

The percentages of alteration for each parameter are shown. Level of statistical significance = *p < 0.05, **p < 0.01, ***p < 0.001. PCC, Post-COVID-19 Condition; RAVLT, Rey Auditory Verbal Learning Test; ROCF, Rey–Osterrieth Complex Figure Test; TMT, Trail Making Test; BNT, Boston Naming Test. Bold values indicate statistical significance.

### 3.3 Moderators between sleep quality and cognition in PCC patients

**Figure 1:**
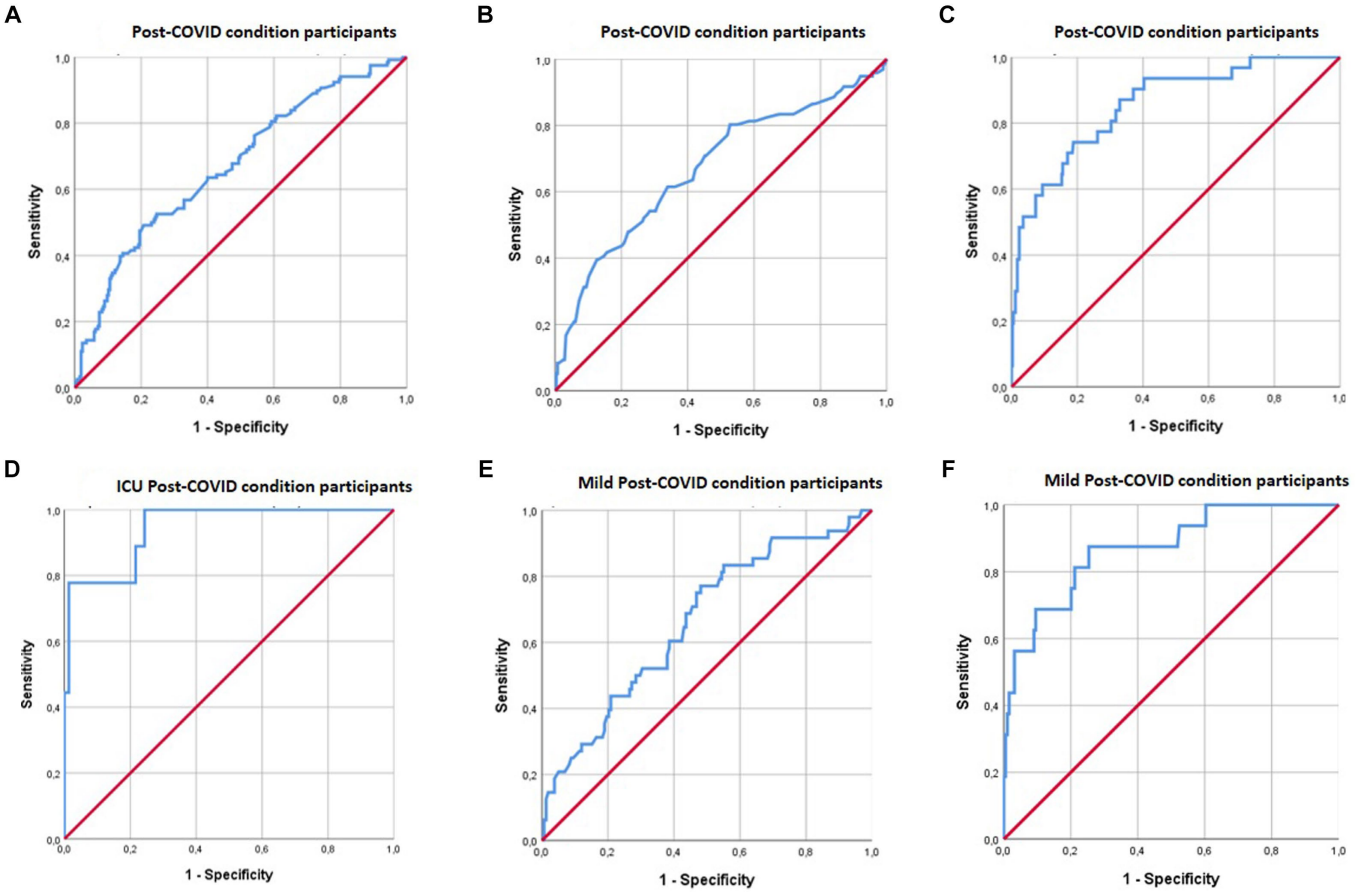
ROC curves for moderators between sleep quality and cognition in PCC patients. **(A)** Shows the combined effect of MFE-30 and EQ-5D moderations on the relationship between sleep quality and RAVLT immediate recall performance. **(B)** Shows the combined effect of CRC moderations on the relationship between sleep quality and ROCF delayed recall performance. **(C)** Shows the combined effect of MFE- 30 and CRC moderations on the relationship between sleep quality and digit symbol performance. **(D)** Shows the combined effect of MFE- 30 and EQ-5D moderations on the relationship between sleep quality and digit symbol performance. **(E)** Shows the combined effect of MFE-30 and EQ-5D moderations on the relationship between sleep quality and ROCF delayed recall performance. **(F)** Shows the combined effect of MFE-30 and CRC moderations on the relationship between sleep quality and digit symbol performance.

To determine whether there were variables that influenced or intervened in the relationship between cognition and sleep quality, we examined whether cognitive reserve, fatigue, daily memory failure and quality of life moderated this relationship. Table 4 and Figure 1 show significant models where only moderation occurred in the relationship between sleep quality and cognition by any of the previously described moderator variables (when the moderator and sleep quality interaction was significant, *p* < 0.05) (Table 4; Figure 1). Among the possible moderators, fatigue (CFQ) was the only factor that did not affect the cognitive performance of participants with COVID-19 regardless of whether the quality of their sleep was good or poor.

**Table 4:** Moderators between sleep quality and cognition in PCC participants.

					Hosmer Lemeshow	AUC (CI 95%)
Factors	*p*	OR	95% C.I. for OR			
			Lower	Upper		
**PCC participants**						
*RAVLT immediate recall*					χ2 (9.193.8) p 0.326	0.671 (0.612.0.730)
MFE-30 x sleep quality	0.001	1,043	1,023	1,063		
EQ-5D x sleep quality	0.008	0.33	0.145	0.748		
*ROCF delayed recall*					χ2 (13.137.8) p 0.107	0.672 (0.605.0.738)
CRC	0.002	0.907	0.853	0.964		
CRC x sleep quality	0.047	0.963	0.929	0.999		
*Digit symbol*					χ2 (4.836.8) p 0.775	0.855 (0.785.0.925)
CRC	0.001	0.769	0.686	0.861		
MFE-30 x sleep quality	0.001	1,059	1,024	1,096		
EQ-5D x sleep quality	0.001	0.021	0.003	0.141		
**ICU PCC participants**						
*Digit symbol*					χ2 (6.374.8) p 0.605	0.944 (0.877.1.000)
CRC	0.004	0.641	0.472	0.869		
MFE-30 x sleep quality	0.015	1,204	1,036	1,398		
EQ-5D x sleep quality	0.016	0.0000246	0.00000000442	0.137		
**Mild PCC participants**						
*ROCF delayed recall*					χ2 (6.521.8) p 0.589	0.662 (0.575.0.750)
MFE-30 x sleep quality	0.018	1,037	1,006	1,068		
EQ-5D x sleep quality	0.011	0.267	0.096	0.74		
*Digit symbol*					χ2 (5.806.8) p 0.669	0.870 (0.777.0.963)
EQ-5D	0.012	0.033	0.002	0.466		
MFE-30 x sleep quality	0.007	1,085	1,022	1,152		
CRC x sleep quality	0.001	0.796	0.695	0.913		

OR, Odds Ratio; AUC, area under curve; PCC, Post-COVID-19 Condition; RAVLT, Rey Auditory Verbal Learning Test; FCRO, Rey–Osterrieth Complex Figure Test; DS, digit symbol; MFE-30, Memory Failures of Everyday Questionnaire; CRC, Cognitive Reserve Questionnaire; EQ-5D, European Quality of Life-5 Dimensions. Bold values indicate statistical significance.

Participants with COVID-19 and poor sleep quality had a 4.3% greater risk of immediate verbal memory deficits (RAVLT immediate recall) and more subjective cognitive complaints (MFE-30) (odds ratio (OR) 1.043; confidence interval (CI) 1.023–1.063). Furthermore, these participants’ risk of immediate verbal memory disorders was multiplied by 2.4 when quality of life (EQ-5D) was low (OR = 0.33; CI = 0.145–0.748).

With delayed visual memory (FCRO delayed recall), people with PCC and poor sleep quality had a 13% lower risk of these alterations with greater cognitive reserve (CRC) (OR = 0.963; CI = 0.929–0.999). If they did not have poor sleep quality, the risk of alterations in delayed visual memory decreased by 9% when cognitive reserve increased.

Finally, with regard to processing speed (DS), people with COVID-19 and poor sleep quality had a 6.7% greater risk of processing speed deficits with more subjective cognitive complaints (MFE) (OR 1.059; CI 1.024–1.096). On the other hand, the risk of slowing processing speed tripled as quality of life decreased (EQ-5D) (OR 0.021; CI 0.003–0.141). If PCC participants did not have poor sleep quality, neither subjective cognitive complaints nor quality of life affected immediate verbal memory performance or processing speed performance.

An analysis of the severity of COVID-19 revealed that participants with mild PCC and poor sleep quality had an 8.5% greater risk of alterations in processing speed with more subjective cognitive complaints (MFE-30) (OR 1.085; CI 1.022–1.152) and a 20% lower likelihood with greater cognitive reserve (CRC) (OR 0.796, CI 0.695–0.913). In both cases, if participants did not have poor sleep quality, the moderator variables did not affect processing speed. Additionally, participants with mild PCC and poor sleep quality tripled (multiplied by 2.9) their risk of alterations in delayed visual memory when they had low quality of life (EQ-5D) (OR 0.267; CI 0.096–0.74). If participants did not have poor quality of sleep, quality of life did not affect visual memory performance. Finally, participants in the ICU-PCC group with poor sleep quality had a 20.4% greater risk of more slowing in processing speed with more subjective cognitive complaints (MFE-30) (OR 1.204; CI 1.036–1.398). Additionally, they had a 65% lower risk of difficulties in the same cognitive domain when they had higher quality of life (EQ-5D) (OR 2.46E-05; CI 4.42E-09-0.137). Again, if the participants in the ICU-PCC group did not have poor sleep quality, subjective cognitive complaints and quality of life did not interfere with processing speed.

## 4 Discussion

Our results showed that sleep quality was significantly worse in PCC patients than in healthy controls and that there are differences in cognitive performance between groups. Participants with mild PCC had more impairments in executive functions (inhibition) and semantic fluency, whereas hospitalized and ICU PCC participants had worse performance in verbal memory. Additionally, ICU participants had more deficits in attention and processing speed tasks, working memory and emotion recognition. Regarding our main aim, we found that there was a relationship between sleep quality and cognition in several domains (verbal and visual memory and processing speed), with a moderating effect of cognitive reserve, quality of life and everyday day memory failure (and, surprisingly, no moderating effect on fatigue). When the PCC participants did not complain of poor sleep quality (PSQI ≥5), there were no changes in cognitive performance or interactions with the moderator variables.

As previously reported, most studies did not find a relationship between sleep quality and cognition. For example, Ozdemir and Tastemur, in a study based on older survivors of COVID-19 (hospitalized and nonhospitalized), reported a positive correlation between sleep quality and depression but not between sleep quality and cognition (Ozdemir and Tastemur, 2023). Similar results were described by Bolattürk and Soylu (2023) in a sample of forty PCC participants, where the PSQI score did not correlate with the MoCA or MMSE score; instead, the MoCA score correlated with the Hamilton Depression Scale score. More examples of the relationship between sleep quality and other variables, such as anxiety, can be found in this study, which also analysed olfactory dysfunction in patients with long COVID (Paranhos et al., 2023). The authors did not find an association between sleep quality and cognition (measured with the MoCA). However, in most of these studies, cognition was assessed only with screening tools such as the MoCA or MMSE, which have some limitations in detecting subtle cognitive impairment. Furthermore, a group of studies analysed the relationship between cognition and other possible symptoms, such as depression, anxiety, quality of life and fatigue, but did not include sleep quality (Woo et al., 2020; Miskowiak et al., 2021; Rousseau et al., 2021; Matias-Guiu et al., 2022).

We found that PCC participants with poorer sleep quality had worse cognitive performance in some domains (verbal and visual memory and processing speed), with a moderating effect on quality of life, cognitive reserve, and everyday memory failure. The only study in which sleep quality explained cognitive dysfunction was the study by Azcue et al. (2022). The purpose of this study was to compare brain fog from PCC with chronic fatigue syndrome. The authors found predictors of cognitive performance according to linear regression analysis: patients’ education explained the highest percentage of variance, whereas sleep quality explained 15.7% of executive function performance (Azcue et al., 2022). However, the authors did not search for the effects of possible moderators, as we did. Our analysis revealed significantly different moderating variables. First, quality of life appeared to be a moderator variable for the performance of processing speed, immediate verbal memory and delayed visual memory tasks when participants had poor sleep quality. However, if PCC participants did not refer to poor sleep quality, they did not show cognitive deficits depending on their quality of life. Similar to our results, Bungenberg et al. reported that attention and processing speed were related to quality of life and excessive daytime sleepiness (measured with the Epworth Sleepiness Scale) but not to sleep quality. Although their cognitive evaluation was also based on extensive neuropsychological assessment, the authors concluded that cognitive performance was not associated with clinical characteristics or with frequently reported symptoms (including sleep problems) (Bungenberg et al., 2022). Recently, quality of life has also been linked to slower mental processing speed, similar to our results, although sleep quality was not the focus of the previous research (Ariza et al., 2023b). However, these previous results confirm our findings on the relationship between sleep quality and processing speed, to which the mediating effect of quality of life may be added.

### 4.1 Sleep quality and cognitive reserve

Another moderator variable that we analysed was cognitive reserve. We assumed that people with higher levels of cognitive reserve would experience less cognitive impairment due to compensatory mechanisms. We found that PCC participants with poor sleep quality had fewer deficits in visual memory and processing speed when their cognitive reserve was greater. If they did not have poor sleep quality, cognitive performance was not affected by cognitive reserve. To our knowledge, there are no previous findings relating cognitive reserve to sleep quality. However, studies have demonstrated the relationship between cognitive performance and cognitive reserve as a possible predictor of future impairment (Costas-Carrera et al., 2022; Devita et al., 2022; Cavaco et al., 2023).

### 4.2 Sleep quality and subjective cognitive complaints

Subjective cognitive complaints were the last possible moderator variable that interfered with cognitive performance. In this case, PCC participants who reported poor sleep quality had more verbal memory and processing speed deficits when they had more complaints about their cognition. However, if they did not have poor sleep quality, their performance in these cognitive domains was not affected by subjective cognitive complaints. Again, few studies have examined the role of subjective cognitive complaints and sleep quality/cognition. Mantovani et al. (2021) studied the prevalence of myalgic encephalomyelitis/chronic fatigue syndrome (ME/CFS) in a sample of SARS-CoV-2 survivors and explored features such as clinical, neuropsychiatric, and neuropsychological profiles. They found that the ME/CFS-like group had worse sleep quality, fatigue, pain, depressive symptoms, and subjective cognitive complaints than those without ME/CFS-like symptoms. However, their sample was not comparable to ours because they focused on this syndrome.

### 4.3 Sleep quality and fatigue

A recent meta-analysis revealed that the proportion of participants who experienced fatigue 12 or more weeks after contracting COVID-19 was 0.32 (Ceban et al., 2022), reinforcing previous findings that demonstrated that fatigue is one of the most prevalent symptoms in post-COVID-19 patients. However, our results showed that fatigue was not a moderating variable when PCC participants had poor quality of sleep in terms of cognitive performance in any of the assessed cognitive domains. Numerous studies have shown a relationship between sleep quality and fatigue and between fatigue and cognition, but no previous studies have shown a relationship between these three variables. Margalit et al. (2022) explored the risk factors for long-term COVID-19 and its possible pathophysiology. They concluded that individuals with long-term COVID-19-related fatigue had poorer sleep quality and a greater proportion of subjective cognitive complaints. However, they did not assess cognition with objective measures but rather from a subjective impression of cognitive decline. The strong positive correlation between fatigue and sleep disturbances is not surprising as significant interactions between sleep, fatigue, and the autonomic nervous system have been described (Tanaka et al., 2015). In a multicentre study, the authors identified factors associated with cognitive impairment and fatigue (Hartung et al., 2022). They performed two multivariate analyses, one with potential predictors in the acute phase of COVID-19 and one with potential predictors from the post-COVID-19 period (which included sleep quality). Only the univariate analysis, not the multivariate analysis, revealed a significant difference in sleep quality and cognition. However, it should be noted that these authors assessed cognition only with the MoCA. Fatigue was a significant predictor of sleep problems.

### 4.4 Future research and limitations

Our findings have potential implications for treatment. We demonstrated that sleep quality may be a trigger for cognitive dysfunction, especially in terms of memory and processing speed, with the moderating effects of quality of life, cognitive reserve, and everyday memory failure. Thus, if sleep quality could be maximized, cognitive impairment in PCC individuals could be reduced. The benefits of different types of interventions for cognitive deficits have been proven. However, we did not find any study that focused on improving sleep quality to minimize its negative effects on other variables.

When interpreting the results, several limitations must be considered. We included only subjective measures of sleep and did not collect information about previous sleep disturbances prior to COVID-19 infection. Instead, we collected information on cognitive performance from an extensive neuropsychological assessment, which enabled us to detect minimal cognitive deficits compared to screening tools. In addition, we have the limitations inherent to a cross-sectional study, such as the impossibility of making causal predictions (cause-effect) and selection bias (as it is a study with consecutive recruitment). The last limitation it is related to the fact that some variables related to the severity of the disease may have not been controlled, like symptoms in the acute phase or its duration. To compensate this, we performed robust statistical analysis to determine the relationships between cognition and sleep quality and between cognition and the moderating effects of other variables, and all the analyses have been adjusted for age, sex, and education.

## 5 Conclusion

In conclusion, our results showed that poor subjective sleep quality is a potential trigger for cognitive deficits. Verbal and visual memory and processing speed were influenced by poor quality of sleep in PCC participants. Quality of life, cognitive reserve and subjective cognitive complaints appeared to be moderator variables. Therefore, implementing therapeutic strategies to maximize sleep quality could reduce sleep disturbances and perhaps reduce cognitive impairment in PCC participants.

## Data availability statement

The raw data supporting the conclusions of this article will be made available by the authors, without undue reservation.

## Ethics statement

The Scientific Ethics Committee of the Hospital Universitari Arnau de Vilanova approved both the study and the consent procedure (CEIC 2384), as did the Drug Research Ethics Committee (CEIm) of Consorci Sanitari de Terrassa (CEIm code: 02-20-107-070) and the Ethics Committee of the University of Barcelona (IRB00003099). The patients/participants provided written informed consent to participate in the study. The studies were conducted in accordance with the local legislation and institutional requirements. Written informed consent was obtained from the individual(s) for the publication of any potentially identifiable images or data included in this article.

## Author contributions

AC-V: Conceptualization, Investigation, Methodology, Project administration, Writing – original draft. GP-R: Conceptualization, Funding acquisition, Writing – review & editing. MA: Writing – review & editing. NC: Methodology, Project administration, Writing – review & editing. BS: Writing – review & editing. CJ: Writing – review & editing. JB: Writing – review & editing. CB: Writing – review & editing. MG: Conceptualization, Funding acquisition, Writing – review & editing.

## Members of the NAUTILUS-project collaborative group

Vanesa Arauzo and Jose A. Bernia, Consorci Sanitari de Terrassa (CST), Terrassa, Spain. Marta Balague-Marmaña and Berta Valles-Pauls, Hospital Sant Joan Despí Moisès Broggi, Consorci Sanitari Integral. Jesús Caballero, Hospital Universitari Arnau de Vilanova, Lleida, Spain. Ester Gonzalez-Aguado and Carme Tayó-Juli, Consorci Sanitari Alt Penedès-Garraf, Vilafranca de Penedés, Barcelona, Spain. Eva Forcadell-Ferreres and Silvia Reverte-Vilarroya, Hospital Verge de la Cinta, Tortosa, Tarragona, Spain. Susanna Forné, Fundació Sant Hospital de la Seu d’Urgell, La Seu d’Urgell, Lleida, Spain. Anna Bartes-Plans and Jordina Muñoz-Padros, Consorci Hospitalari de Vic, Vic, Barcelona, Spain. Jose A. Muñoz-Moreno and Anna Prats-Paris, Servei de Malalties Infeccioses, Fundació Lluita contra les Infeccions – Hospital Universitari Germans Trias i Pujol, Badalona, Barcelona, Spain. Inmaculada Rico and Nuria Sabé, Hospital Universitari de Bellvitge, L’Hospitalet de Llobregat, Barcelona, Spain. Marta Almeria and Laura Casas, Hospital Universitari Mútua Terrassa, Terrassa, Barcelona, Spain. Maria José Ciudad and Anna Ferré, Badalona Serveis Assistens, Badalona, Barcelona, Spain. Tamar Garzon and Manuela Lozano, Institut d’Assistència Sanitària, Girona, Spain. Marta Cullell and Sonia Vega, Fundació Salut Empordà, Figueres, Girona, Spain. Sílvia Alsina, Fundació Hospital de Puigcerdà, Puigcerdà, Girona, Spain. Maria J. Maldonado-Belmonte and Susana Vazquez-Rivera, Hospital Universitario Central de la Cruz Roja San José y Santa Adela, Madrid, Spain. Eva Baillès and Sandra Navarro, Servei Andorrà d’Atenció Sanitària (SAAS), Andorra. Ayoze González Hernández, Facultad de Ciencias de la Salud, Universidad Fernando Pessoa Canarias. Yaiza Molina, Clínica Universitaria de Psicología, Facultad de Ciencias de la Salud, Universidad Fernando Pessoa Canarias. Victoria Olive, Occupational Health Care Service, Hospital Clínic Barcelona. Silvia Cañizares, Section of Clinical Psychology of Health, Clinical Institute of Neurosciences, Hospital Clinic of Barcelona. Department of Clinical Psychology and Psychobiology, Universitat de Barcelona.
